# Epigenetic Remodeling in Thyroid Cancer: New Dimensions of Targeted Therapy Through lncRNA Modulation

**DOI:** 10.3390/cimb47100863

**Published:** 2025-10-18

**Authors:** Adrian Albulescu, Alina Fudulu, Mirela Antonela Mihaila, Iulia Iancu, Adriana Plesa, Marinela Bostan, Anca Botezatu, Lorelei Irina Brasoveanu, Camelia Mia Hotnog

**Affiliations:** 1 Molecular Virology Department, Stefan S. Nicolau Institute of Virology, Romanian Academy, 030304 Bucharest, Romania; adrian.albulescu@virology.ro (A.A.); iulia.iancu@virology.ro (I.I.); adriana.plesa@virology.ro (A.P.); anca.botezatu@virology.ro (A.B.); 2 Pharmacology Department, National Institute for Chemical Pharmaceutical Research and Development, 031299 Bucharest, Romania; 3 Center of Immunology, Stefan S. Nicolau Institute of Virology, Romanian Academy, 030304 Bucharest, Romania; mirela.mihaila@virology.ro (M.A.M.); marinela.bostan@virology.ro (M.B.); camelia.hotnog@virology.ro (C.M.H.)

**Keywords:** lncRNAs, epigenetics, thyroid cancer, K1 human cell line, methylation, apoptosis, cell cycle, cytokine release

## Abstract

Thyroid carcinomas are phenotypically heterogeneous malignancies. Advances in molecular and cellular technologies have revealed genetic, epigenetic, and nongenetic factors underlying this heterogeneity. Our study aimed to assess the impact of single and combined treatments with anticancer agents (Carboplatin, Doxorubicin, Paclitaxel, Avastin), natural compounds (Quercetin), and epigenetic modulators (suberoylanilide hydroxamic acid and 5-Azacytidine) on the expression of long noncoding RNAs, methylation regulators, and functional features in the human thyroid cancer cell line K1. Methods: Treated and untreated K1 cells were used throughout experiments to evaluate the drug-induced cytotoxicity, apoptosis, cell cycle distribution, cytokine release, gene expression, and global DNA methylation levels. Results: Some single- and combined-drug treatments modulated both cell cycle progression and apoptotic events, demonstrating anti-tumor activity of the tested compounds. Gene expression analysis showed treatment-specific regulation of target genes and lncRNAs, including both upregulation and downregulation across different drug combinations. All treatments resulted in increased global DNA methylation levels compared to the untreated controls. Several combinations significantly upregulated DNMT1 and DNMT3B, while concomitantly decreased EZH2 levels. Conclusions: These coordinated epigenetic changes highlight the therapeutic potential of combining epigenetic modulators with chemotherapeutic agents, suggesting a strategy to prevent or reverse treatment resistance and improve outcomes in thyroid cancer patients.

## 1. Introduction

Thyroid cancer, ranked the seventh in the absolute incidence among cancers [1,2], is the most common malignant endocrine disease, affecting both genders, although it is more common in women, with rates up to three to four times higher than in men [3]. Thyroid cancer is highly heterogeneous with diverse cytological, histological, and genetic features. Approximately 90% of thyroid cancers are differentiated thyroid cancers (DTC) [4], which include papillary thyroid cancer (PTC) and follicular thyroid cancer (FTC) subtypes. Medullary thyroid carcinomas (MTC) comprise 5–10% of thyroid cancer cases, while anaplastic thyroid carcinomas (ATC) account for up to 1–2% [5,6]. Although the prognosis for most differentiated thyroid cancers remains favorable [7], patients over 60 years of age have lower disease-free and disease-specific survival rates [8], with ATC exhibiting a highly aggressive behavior, having limited treatment options, and significant resistance to conventional therapies [9]. In this context, exploration of epigenetic mechanisms in thyroid oncogenesis has gained an increasing attention, given their potential roles in tumor progression, therapeutic response, and patient prognosis.

The scientific literature has established that the majority of RNA transcripts in cells do not encode proteins. Instead, noncoding RNA species, such as microRNAs, long noncoding RNAs (lncRNAs), and circular RNAs are more abundant than their protein-coding counterparts [10,11]. These molecules are deeply involved in cancer processes, including cell proliferation, resistance to apoptosis, invasion, metastasis, and genomic instability. In particular, lncRNAs have been shown to display aberrant expression profiles in malignancies, closely correlating with the processes of tumor initiation and progression [12]. Notably, lncRNAs often exhibit high cell type and tissue specificity, distinguishing them from more ubiquitously expressed transcription factors or coding genes. This unique expression signature highlights their potential as precise, personalized therapeutic targets in the emerging landscape of molecular oncology [13].

Classical anticancer therapeutic approaches in various types of solid tumors usually involve treatment with cytostatic drugs, used alone or in combination with other therapeutic agents. Among the most commonly used drugs in the treatment of thyroid cancer are the chemotherapeutic agents like doxorubicin, taxanes (paclitaxel or docetaxel), and platinum ion compounds (cisplatin or carboplatin). However, chemotherapy has a limited role in patients with all subtypes of thyroid cancers, having poor response rates, short duration of response, and undesirable toxic side effects [14,15]. Doxorubicin/Adriamycin (Dox) has antiproliferative effects regardless of the cell cycle phase of the cell [16], and has been shown to inhibit growth in human thyroid cell lines K1, Cal-62, 8505C, and 8305C, when used in combination with bioactive compounds (curcumin) or epigenetic modulators (GSK-J4) [17,18].

Paclitaxel (Pxl) is widely used in cancer therapy due to its ability to stabilize and prevent microtubules depolymerization, leading to cell cycle arrest in the G2/M phase and promoting cell death [19,20]. Paclitaxel has been shown to inhibit the cell viability of ACT-1, 8305c, and 8505c cell lines, derived from anaplastic thyroid carcinoma, in a dose-dependent manner [21].

Carboplatin (CPt) is one of the main platinum-based anti-tumor drugs that inhibits replication and transcription, and induces programmed cell death [22,23].

In recent years, immunotherapeutic approaches, such as targeted monoclonal antibody treatments, have been extensively used in oncology departments. Among them, Avastin/Bevacizumab (Ava), an anti-angiogenic monoclonal antibody, targets and binds to vascular endothelial growth factor (VEGF) and exerts its biological activity without significant cytotoxic effects [24]. In addition, biologically active natural compounds have gained attention for their anticancer properties, including antioxidant, immunomodulatory, and apoptosis-inducing effects. Quercetin (Qct), a plant-derived flavonoid, has demonstrated anti-tumor activities along with a low toxicity profile, making it a promising candidate for integrative therapeutic strategies [25].

PTC is generally associated with a favorable prognosis; however, a subset of patients has shown recurrence and resistance to conventional therapies, highlighting the need for improved treatment strategies. The human thyroid cancer cell line K1 (ECACC, 92030501), derived from a metastatic PTC, serves as a valuable in vitro model for investigating molecular mechanisms underlying thyroid oncogenesis and for evaluating novel therapeutic interventions.

A series of lncRNAs known to be involved in various mechanisms related to cancer pathology were selected for this study. While EMX2OS predicts shorter recurrence-free survival for PTC [26], ZFAS1 has been shown to promote PTC progression through sponging of miR-590-3p and upregulating of HMGA2 [27]. TUG1 is involved in proliferation, migration and epithelial–mesenchymal transformation by targeting miR-145 [28]. MALAT1 has oncogenic roles in multiple types of cancer [29,30]. H19 is known to be downregulated in thyroid cancer samples compared to controls, and its lower expression is correlated with lymph node metastasis; reactivation of its expression may lead to the inhibition of tumorigenesis and could serve as a prognostic and diagnostic tool [31]. MEG3 also has tumor suppressor activity, enhancing apoptosis and cellular sensitivity to treatments [32]. HOTTIP is a known lncRNA with oncogenic activity, promoting proliferation, invasion and migration [33]. GAS5 functions as a competitive endogenous RNA that regulates PTEN expression by sponging miR-222-3p, being generally under-expressed in PTC samples [34]. NRON and HAR1B are under-investigated in PTC and require further study.

Considering all the above, the present study aims to demonstrate whether treatments with epigenetic modulators, single or combined with conventional chemotherapeutic agents, targeted monoclonal antibodies, and/or natural compounds might bring some benefit in treating thyroid cancer. Therefore, epigenetic modulators such as histone deacetylase inhibitor (HDACi) suberoylanilide hydroxamic acid (SAHA) [35,36] and DNA methyltransferase inhibitor 5-Aza-Cytidine (5-Aza-C), individually or in combination with CPt, Dox, Pxl, Ava, or Qct, were investigated for their effects following the treatment of K1 human thyroid cancer cells. We evaluated the potential modulation of the expression levels of selected lncRNAs’ (NRON, EMX2OS, ZFAS1, HAR1B, TUG1, MALAT1, H19, MEG3, HOTTIP, GAS5, HOTAIR) and methylation-related genes (EZH2, DNMT1, DNMT3A, and DNMT3B), along with the investigation of cytotoxic effects and impact on apoptosis, cell cycle distribution, and cytokine release.

## 2. Materials and Methods

### 2.1. Reagents, Drugs, and Kits

Suberoylanilide hydroxamic acid (SAHA), 5-Aza-Cytidine (5-Aza-C), Doxorubicin (Dox), Quercetin (Qct), ethylenediaminetetraacetic acid (EDTA), dimethyl sulfoxide (DMSO), phosphate-buffered saline (PBS), and paraformaldehyde (PFA) were purchased from Sigma Aldrich (St. Louis, MO, USA). Avastin (Ava) was acquired as a 25 mg/mL Bevacizumab concentrate for infusion from Roche Reg. Ltd. (Welwyn Garden City, UK), while Carboplatin (CPt) was obtained as a 10 mg/mL concentrate for infusion from Teva Ltd. UK (Eastbourne, East Sussex, UK). Paclitaxel (Pxl) was purchased as a 6 mg/mL concentrate for infusion from Fresenius Kabi AG (Bad Homburg, Germany).

The Dulbecco’s Modified Eagle’s Medium was purchased from PAN Biotech, Aidenbach, Germany, while fetal bovine serum (FBS), 200 mM L-Glutamine, 100× concentrated antibiotic mixture (10,000 U/mL penicillin and 10,000 µg/mL streptomycin) were provided from Biochrom GmbH, Berlin, Germany.

CellTiter 96^®^ AQueous One Solution Cell Proliferation Assay (MTS) kit, provided by Promega, Madison, WI, US, was used in the cytotoxicity assays. FxCycle PI/RNase Staining Solution (Thermo Fisher Scientific by Invitrogen Life Technologies Corporation, Hillsboro, OR, US) was used for cell cycle phases analysis, while the Annexin-FITC Apoptosis Detection kit (Becton Dickinson (BD) Biosciences, Mountain View, CA, USA) served for the evaluation of cell apoptosis. Levels of soluble cytokines were measured by using the human IL-6 and TNF-α uncoated ELISA kits (Invitrogen, Bender MedSystems GmbH, Vienna, Austria).

### 2.2. In Vitro Cell Culture and Treatment Conditions

The K1 human thyroid cancer cell line, derived from the primary papillary thyroid GLAG-66 carcinoma cell line, was obtained from the European Collection of Authenticated Cell Cultures (ECACC, 92030501). The cells were cultured following the provider’s guidelines in a DMEM: Ham’s F12: MCDB 105 (2:1:1) medium, supplemented with 2 mM L-Glutamine and 10% FBS, at 37 °C in a 5% CO_2_ humidified atmosphere.

When grown in 25 or 75 cm^2^ flasks and reaching approximately 60–70% confluence, the cells were treated for 24–48 h with various concentrations of epigenetic modulators (SAHA, 5-Aza-C), oncolytic drugs (Dox, CPt, Pxl), natural compounds (Qct), or monoclonal antibodies (Ava). Following treatments, cells were detached using a non-enzymatic PBS/1 mM EDTA solution, washed twice in PBS, and either immediately subjected to compound-mediated cytotoxicity assays, apoptosis evaluation via flow cytometry, or preserved for nucleic acid extraction for molecular biology analyses. For cell cycle analysis by flow cytometry, cells were fixed in ice-cold ethanol/PBS (70:30) and stored at 4 °C until use. In addition, cell culture supernatants were collected, centrifuged at 400× *g*, and stored at −80 °C for subsequent soluble cytokine evaluation. Non-treated (NT) cells served as controls in all experiments [37,38].

### 2.3. Cell Viability Testing and Cytotoxic Effects

To explore the potential of epigenetic modulators (SAHA), oncolytic drugs (CPt, Pxl, Dox), and bioactive compounds (Qct) in enhancing the chemosensitivity and counteracting drug resistance, their impact on cell proliferation was evaluated in the K1 thyroid cancer cell line. Cytotoxicity was assessed using the MTS [3-(4,5-dimethylthiazol-2-yl)-5-(3-carboxymethoxyphenyl)-2-(4-sulfophenyl)-2H-tetrazolium] assay, a colorimetric method for measuring the cell viability. All experiments were performed in triplicate using the CellTiter 96^®^ AQueous One Solution Assay. The assay measures the cell viability based on the MTS-to-formazan conversion, enhanced by the cationic dye PES (phenazine methosulfate), with the absorbance measured at λ = 492 nm.

For this, 10^4^ cells were seeded in 100 µL of culture medium per well in 96-well flat bottom microtiter plates and incubated for 24 h to allow cell adhesion. After removing the culture supernatants, cells were treated for 24–48 h with increasing concentrations of CPt, SAHA, and Qct (6.25, 12.5, 25, 50, 100, and 200 µM), Pxl (0.125, 0.25, 0.5, 1, 2, and 4 µM), or Dox (1.25, 2.5, 5, 10, 20, and 40 µM). Subsequently, 20 µL of the MTS reagent mixture was added to each well and the plates were incubated for 4 h at 37 °C with mild shaking every 15 min, absorbance was measured at 492 nm using a Dynex ELISA reader (DYNEX Technologies—MRS, Chantilly, VA, USA) [39].

Cell viability was expressed as a percentage relative to untreated control cells (NT), which were set as the 100% viability reference, and was calculated using the following formula:

Cell viability (%) = 100 × (T − B)/(U − B)

Then, to better express the cytotoxic effects, results were presented as percentages of cell lysis:

Cell lysis (%) = 100% − Cell viability (%)

where T represents the optical density of treated cells, B is the optical density of the blank (culture medium without cells), and U is the optical density of untreated cells.

The experiments were performed in triplicate, and the results were reported as mean values ± standard deviation (SD) (n = 3). To exclude the nonspecific MTS interactions with SAHA, Dox, CPt, Pxl, and Qct, the absorbances of all tested drug concentrations were spectrophotometrically measured in the absence of the cells and subtracted during calculations.

### 2.4. Real-Time Cell Analysis (RTCA)

The balance between cytotoxicity and cell proliferation was also assessed by Real-Time Cell Analysis (RTCA) technique that allows a continuous monitoring of the cellular behavior using several characteristics of the cells such as adhesivity and morphology. Valuable information regarding changes in cell proliferation versus cytotoxicity, due to cell cycle arrest or senescence, could be achieved. This label-free method continuously monitors the cell activity using the xCELLigence DP System (ACEA Biosciences, San Diego, USA) and specialized E-Plates. Each well contains integrated microelectrodes that measure the electrical impedance in real time, generating cell index (CI) values that are proportional to the number of viable cells [40].

For the assay, 10^4^ of K1 cells were seeded per well in E-Plate 16 plates that were placed inside the xCELLigence DP System, housed in a humidified incubator with 5% CO_2_. The system, connected to a computer and operated via the RTCA 2.1.2 software, automatically started to record the growth curves, this time-point being registered as the start point, when CI is equal to 0. The results were presented as normalized cell indexes (CI) after the automatic comparison between the curves of viability for treated and non-treated cells, the time-point chosen for the normalization being shown by a black vertical marker, fixed for the time-point of adding treatments (approximately 24 h of cell growth). When cell growth and normalized CI decreased under the baseline level (drawn as a black horizontal dotted line for normalized CI = 0.3), the continued monitoring of proliferation and cytotoxic responses was stopped; this time-point is considered the end-point of the experiment.

### 2.5. Flow-Cytometric Evaluation of Cell Cycle Distribution and Apoptosis

The distribution of K1 cell populations in different cell cycle phases was evaluated by flow-cytometry technique. Briefly, a total of 10^5^ of K1 thyroid cancer cells, either non-treated (NT) or treated for 48 h with epigenetic modulators (5 µM SAHA or 5-Aza-C), and/or other compounds, including the oncolytic drugs CPt (50 µM), Dox (0.5 µM), Pxl (1 µM), the anti-VEGF monoclonal antibody Ava (20 µg/mL), and the natural compound Qct (50 µM), previously fixed and preserved as described in Section 2, were twice washed with cold PBS and centrifuged for 5 min at 300× *g* at 4 °C. Then, cells were treated with 0.5 mL of FxCycle™ PI/RNase Staining Solution and incubated for 10–30 min in the dark at room temperature (RT). For cell cycle assessment 30,000 events were acquired on a FACS CANTO II flow cytometer (BD Biosciences, Immunocytometry System, Mountain View, CA, US) using DIVA 6.2 software. The nuclear DNA content in nuclei and progression through cell cycle phases was analyzed using ModFIT^LT^ 2.3 software [41].

The potential induction or modulation of apoptosis in K1 cells were also investigated by flow cytometry. One of the most used experimental approaches is Annexin V-FITC/PI double staining of cells, followed by flow-cytometry acquisition, and further analyses of single- or double-labeled cells. Untreated and 48 h-treated cells were detached with PBS/1 mM EDTA, twice washed with PBS, centrifuged for 5 min at 300× *g*, and cell pellets resuspended in 400 µL binding buffer. Then, 10^5^ cells were stained with 5 µL of Annexin-V/FITC and/or PI per 100 µL binding buffer/tube and incubated for 15 min/ RT in the dark; 10,000 events per sample were acquired using the FACS CANTO II flow cytometer, apoptotic cells were analyzed using DIVA 6.2 software and quantified as percentages [42]. The assay allows discrimination between the viable cells (FITC^−^/PI^−^) and necrotic cells (FITC^−^/PI^+^), and the evaluation of both early (FITC^+^/PI^−^) and late (FITC^+^/PI^+^) apoptosis.

The experiments were performed in triplicate, and the results were reported as mean values ± standard deviation (SD) (n = 3).

### 2.6. ELISA-Based Detection of Cytokines

To quantify the release of several cytokines in the cell culture supernatants of treated versus untreated K1 thyroid cancer cells, we performed several Enzyme-linked Immunosorbent Assay (ELISA) tests for the quantitative detection of human IL-6 and TNF-α, using uncoated ELISA kits. Briefly, 96-well plates were coated with capture antibodies and incubated overnight at 4 °C. After washings with 200 µL of 0.05% PBS-Tween 20 buffer, 100 µL of standard dilutions or supernatant samples were added, followed by sequential detection with specific antibodies, streptavidin–HRP, and TMB substrate. The reaction was stopped with (NH_4_)_2_SO_4_, and absorbance was spectrophotometrically measured at λ = 450 nm. Cytokine concentrations were calculated from standard curves (starting from 500 pg/mL for IL-6, and 1000 pg/mL for TNF-α) and expressed in pg/mL. The results were normalized versus the untreated controls, considered 100% [43]. The experiments were performed in triplicate, and the results were reported as mean values ± standard deviation (SD) (n = 3).

### 2.7. Quantification of Genes and lncRNAs Expression by qRT-PCR

Total RNA was isolated and purified from all treated and untreated samples of K1 human thyroid cancer cell line using the RNeasy Mini Kit (Qiagen, Hilden, Germany) according to the manufacturer’s protocol, then reverse-transcribed into cDNA using the High-Capacity cDNA Reverse Transcription Kit (Thermo Fisher Scientific, Waltham, MA, US) and 1 µg of RNA per sample was used as input [44,45].

mRNA expression levels were quantified by qRT-PCR using the Applied Biosystems 7300 Real-Time PCR System (Foster City, CA, USA), with U6 serving as the reference gene. Each reaction mixture had a final volume of 25 µL and contained 12.5 µL of Maxima SYBR Green/ROX qPCR Master Mix (2×) (Thermo Fisher Scientific, Waltham, MA, USA), 0.30 µM of each primer, and 5 µL of cDNA (10 ng/µL). The relative expression of target genes was investigated in triplicate samples from treated and untreated K1 cells. Data were calculated using either the 2^−∆Cq^ or 2^−ΔΔCq^ method and expressed as mean log10 values ± SD. Primer sequences were established in our previous studies and listed in the Supplementary Table S1 [45].

### 2.8. Genomic DNA Extraction and LINE-1 Methylation Profiling

DNA was isolated from treated and untreated cells using the QIAamp DNA Mini Kit (Qiagen, Hilden, Germany) according to the manufacturer’s protocol. DNA concentration and purity were measured with the NanoDrop ND-1000 spectrophotometer (Thermo Fisher Scientific, Waltham, MA, USA).

LINE-1 methylation was assessed using an ELISA-based assay for detecting 5-methylcytosine (5-mC) levels in genomic DNA, using the Global DNA Methylation LINE-1 ELISA kit (Active Motif, Carlsbad, CA). Extracted genomic DNA was digested ON/ 37 °C with 10 U/μL at MseI, hybridized to a biotinylated LINE-1 probe targeting CpG-rich regions of the LINE-1 repeat motif, and incubated with 5-mC specific antibodies conjugated with HRP on streptavidin-coated plates. Absorbance was measured in triplicate samples at λ = 450 nm (reference wavelength, λ = 655 nm) using a TriSta^r2^S microplate reader (Berthold Technologies, Bad Wildbad, Germany). Methylation levels were expressed as the percentage of 5-mC relative to total cytosine, with assay standards provided alongside the samples.

### 2.9. Statistical Analysis

Statistical analysis was performed using GraphPad Prism version 10.3 (GraphPad Software Inc., San Diego, CA, USA) on triplicate samples, with significance for *p*-values < 0.05. Gene and lncRNA expression data were presented as mean log10 values ± standard deviation (SD) and analyzed using the Mann–Whitney test or Student’s *t*-test, as appropriate. Pearson’s test was used for correlation analysis of *n*-fold gene expression data. Cytotoxicity, apoptosis, and ELISA results were presented as mean values ± SD, and evaluated using Student’s *t*-test or one-way ANOVA. Pearson’s tests were used for correlation analysis between the various investigated parameters.

## 3. Results

### 3.1. Evaluation of Dose- and Time-Dependent Compound-Induced Cell Cytotoxicity

**Figure 1:**
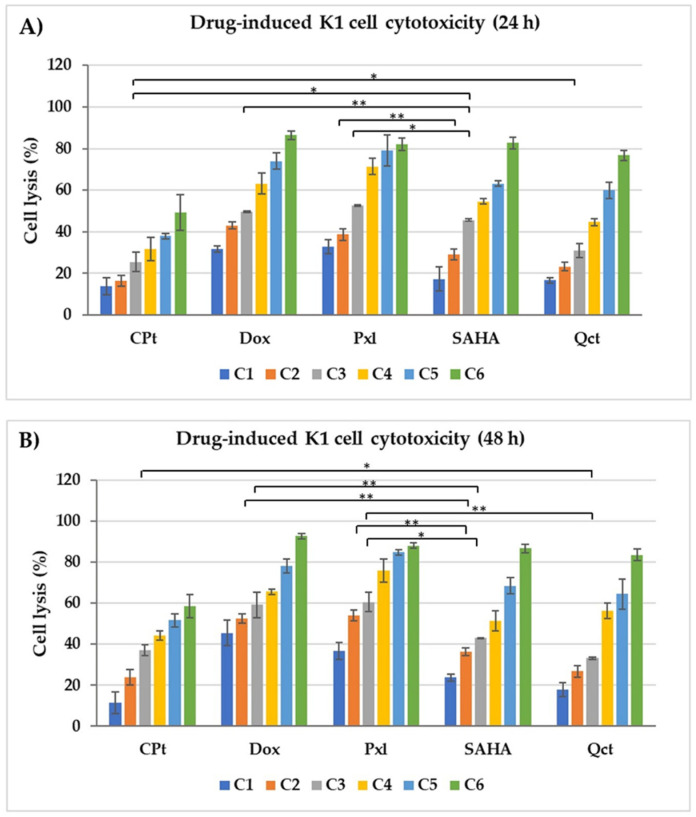
The cytotoxic effects of six scalar increasing concentrations (C1–C6) of CPt, Dox, Pxl, SAHA, and Qct on K1 human thyroid cancer cells, expressed as cell lysis percentages. Panel (**A**): 24 h exposure; panel (**B**): 48 h exposure. Data were expressed as mean values ± SD (n = 3) (* *p* < 0.05, ** *p* < 0.01).

The percentages of cell lysis were modulated in various ways by the compound treatments, as shown in Figure 1, both for 24 h and 48 h adding of scalar concentrations of drug treatments. Cell responses to drug treatments presented an increase in cell lysis, and implicitly a decrease in cell proliferation (or viability), which depended on both drug concentrations and duration of treatments, with stronger effects being observed for 48 h compared to 24 h treatments (Figure 1, panel A and B). Treatments applied for 24 h with 50, 100, and 200 µM of CPt increased the K1 cell lysis up to 31.83%, 38%, and 49.35%. The same concentrations of SAHA or Qct had a stronger effect: the percentages of cell lysis increased up to 54.63%, 63.12%, and 82.72% for SAHA, and up to 44.71%, 60%, and 76.84% for Qct treatments. The 12.5 and 25 µM treatments with the same drugs increased cell lysis to 16.51% and 25.48% for CPt, to 29.1% and 45.61% for SAHA, and to 23.22% and 31.18% for Qct (Figure 1A).

Strong effects were also observed when Dox or Pxl were used. The higher concentrations of 40, 20, and 10 μM of Dox increased the cell lysis percentages up to 86.4%, 74%, and 63.24%, respectively, while 4, 2, and 1 μM of Pxl induced the increase in cell lysis up to 82.19%, 79.16, and 71.38%. Lower concentrations of the two drugs also had cytotoxic effects: 2.5 and 5 μM of Dox induced cell lysis levels of 43.11% and 49.58%, while 0.25 and 0.5 μM of Pxl induced 38.72% and 52.61% of cell lysis (Figure 1, panel A).

Statistical analyses showed significant differences in levels of cell lysis when different treatments and various concentrations were compared. When C3 concentrations for each compound were compared, we observed significant differences between the treatments with 50 µM CPt vs. 12.5 µM SAHA (*p* = 0.0412), 50 µM CPt vs. 50 µM Qct (*p* = 0.0437), 10 µM Dox vs. 12.5 µM SAHA (*p* = 0.0065), and 1 µM Pxl vs. 12.5 µM SAHA (*p* = 0.0135). For C2 concentrations, significant data were obtained between 2 µ M Pxl and 25 µM SAHA treatments (*p* = 0.0023) (Figure 1, panel A).

In addition to the dose-dependent effects, extending drug exposure to 48 h further increased cell lysis in K1 thyroid cancer cells (Figure 1B). For CPt, concentrations of 12.5 to 200 µM induced 23.93 to58.57% cell lysis. Stronger effects were observed with SAHA (36.41 to 86.69%) and Qct (26.86 to 83.57%) across the same concentration range. Dox treatments with 1.25 to 40 µM increased cell lysis from 45.48% to 92.69%, while prolonged exposure to Pxl (0.125 to 4 µM) elevated lysis from 36.64% to 88% (Figure 1B).

We have also performed statistical analyses for the results obtained after 48 h of treatments and we observed similar behavior as for 24 h. For example, when C3 concentrations were compared, we obtained significant differences between 2 µM Pxl vs. 25 µM SAHA (*p* = 0.0018), 20 µM Dox vs. 25 µM SAHA (*p* = 0.0092), 50 µM CPt *vs*. 50 µM Qct (*p* = 0.0427), 10 µM Dox *vs*. 12.5 µM SAHA (*p* = 0.0198), 1 µM Pxl vs. 12.5 µM SAHA (*p* = 0.0490), and 1 µM Pxl vs. 50 µM Qct (*p* = 0.0015).

From the results obtained after performing the MTS assay, expressed as mean values of cell lysis percentages ± SD, we calculated the values of drug concentrations necessary to inhibit 50% (IC50) and 75% (IC75) of cell growth for each tested compound (Table 1).

### 3.2. RTCA Continuous Monitoring of Single vs. Combined Treated K1 Cells

**Figure 2:**
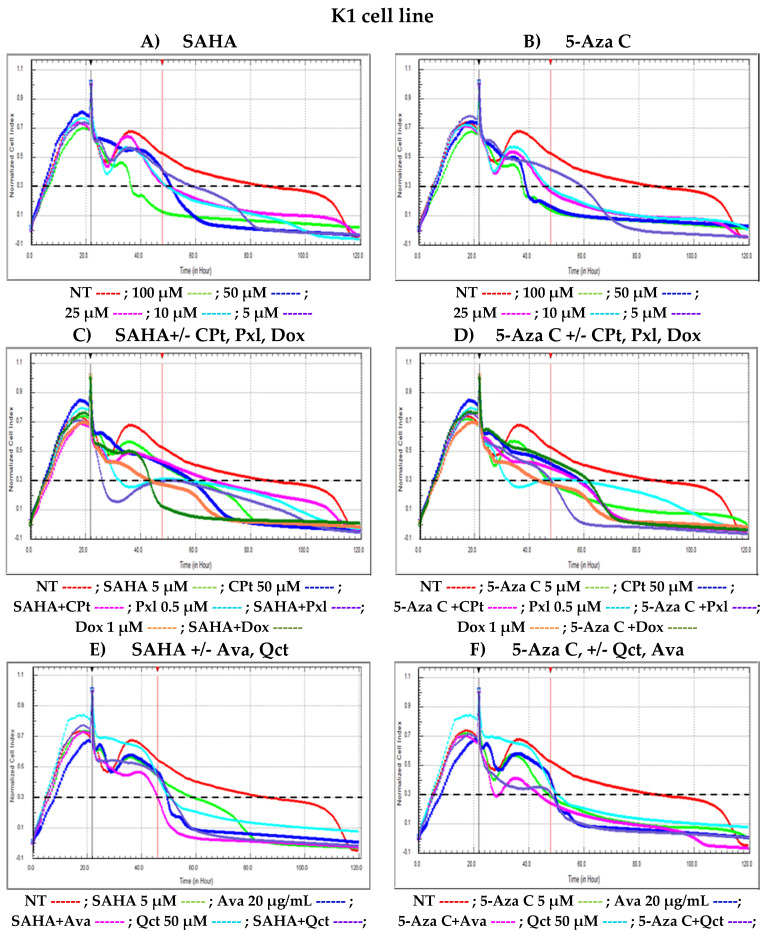
RTCA label-free monitoring of single and combined SAHA or 5-Aza-C mediated cytotoxicity of treated K1 thyroid cancer cells by xCELLigence Real-Time Cell Analyzer. After approximately 24 h of cell cultures in E-16 plates, single or combined SAHA or 5-Aza-C treatments were added. Following the automatic registration of the entire culture and treatment process, results were presented as normalized cell indexes (CI) after the automatic comparison between the viability curves for treated versus non-treated cells (NT). The black vertical marker was fixed for the time-point when treatments were added and the normalization was made; the red vertical marker represents the time-point chosen for a particular RTCA analysis. The black horizontal dotted line was chosen as baseline for the normalized cellular index equal to 0.3.

The results were expressed as normalized cell indexes between viability growth curves of treated versus non-treated cells (Figure 2).

When single treatments with either SAHA or 5-Aza-C were applied to K1 cells in culture, a significant decrease in cell proliferation was observed, normalized cellular indices (CI) diminished in a dose- and time-dependent manner suggesting cytotoxic properties of these epigenetic agents. The growth curves presented in Figure 2, panels A and B show the ability of different concentrations of these epigenetic regulators to induce lysis of K1 cells. Their combinations with either chemotherapeutic agents such as CPt, Pxl, Dox (Figure 2, panels C and D), monoclonal antibodies like Ava or natural compounds such as Qct (Figure 2, panels E and F), seem to increase the levels of cell lysis compared to single treatments since CI values decreased compared to NT cells.

The results obtained after performing the cytotoxicity tests, either the MTS end-point colorimetric assay, or the continuously monitored RTCA experiment led us to choose several fixed concentrations to be further used in molecular biology assays, or various biological approaches like modulation of cell apoptosis, cell cycle phases, and cytokine release in cell culture supernatants. Therefore, K1 human thyroid cancer cells were treated for 48 h with fixed concentrations of 50 μM for CPt and Qct, 0.5 μM for Pxl, 0.5 μM for Dox, and 5 μM for SAHA.

For 5-Aza-C epigenetic drug, we chose the concentration of 5 µM, based on our previous findings [44]. In addition, Ava monoclonal antibody was used at 20 µg/mL, a dose shown to effectively bind VEGF and exert its biological activity without significant cytotoxic activity, despite being lower than the therapeutic dose used in patients [24].

### 3.3. Modulation of Cell Cycle Phases and Apoptosis in K1 Thyroid Cancer Cells

**Figure 3:**
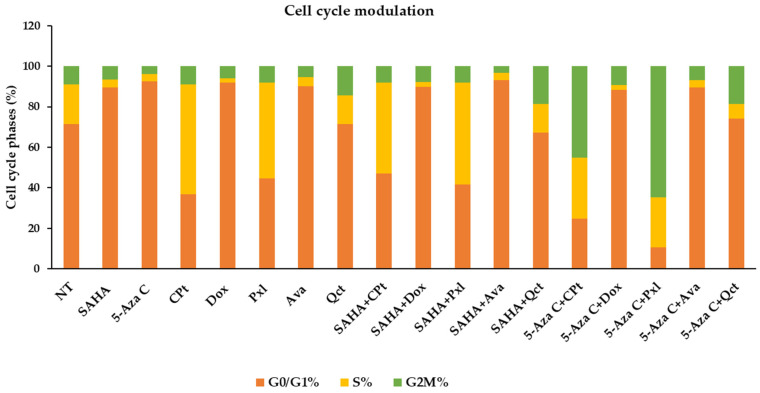
DNA distribution through K1 cell cycle phases after exposure to single or combined SAHA or 5-Aza-C treatments. G0/G1 phase is indicated in orange, the S phase is colored yellow, while G2/M phase is represented by the green region.

Both cell cycle phases and apoptotic events can be modulated by single- or combined-drug treatments, thus showing the anti-tumor effects of the compounds under study. When non-treated control cells were analyzed for the nuclei distribution in cell cycle phases, we observed a cell distribution of 71.41% in G0/G1, 19.7% in S, and 8.89% in G2M cell cycle phases in a representative experiment. Figure 3; Supplementary Materials—Table S2, Figure S1.

Single treatments of the K1 cell cultures with SAHA, 5-Aza-C, Dox, Ava, or Qct induced a decrease in nuclei percentages in S phase until 3.68%, 3.71%, 2.28%, 4.65%, and 14.18%, respectively. SAHA, 5-Aza-C, Dox, and Ava treatments have also increased the cell levels in G0/G1 phase to 89.69%, 92.57%, 91.85%, and 90.16%, respectively, while Qct induced an increase in the nuclei percentages in G2M phase until 14.24%. In contrast, Pxl and CPt treatments decreased the G0/G1 phase to 44.56% and 36.66%, and therefore increased the proliferation indexes (S% + G2M%) to 55.44% and 63.34%, respectively (Figure 3, Supplementary Materials—Table S2, Figure S1).

When we analyzed the effects of combined treatments of SAHA or 5-Aza-C epigenetic drugs on K1 cells, we observed nuclei increases in G0/G1 phase when administered as single treatments or combined with Dox or Ava. In addition, decreases in S phase were observed with SAHA or 5-Ava-C administered alone, and the decreases were even stronger when combined with Dox. On the contrary, treatments with CPt or Pxl as single agents led to increased S phase and the association with 5-Aza-C decreased the rate of S phase but remained higher compared with the NT control. These combination drug therapies induced a strong increase in G2M phase, to 45.25%, and 64.59%, respectively, and of the proliferation indexes to 75.3% and 89.51% (Figure 3, Table S2, Figure S1).

Another biological effect of drug treatments consists of the modulation of cell apoptosis. Annexin V is a phospholipid-binding protein that binds to phosphatidylserine, and is calcium dependent, and thus allows the identification of apoptotic cells, either found in early (Annexin-FITC^+^PI^−^/quadrant Q4) or late (Annexin-FITC^+^PI^+^/quadrant Q2) stages. Double negative cells count for viable cells (quadrat Q3), while single PI-colored cells are considered necrotic cells (quadrant Q1) (see representative dot plots in Figure S2).

**Figure 4:**
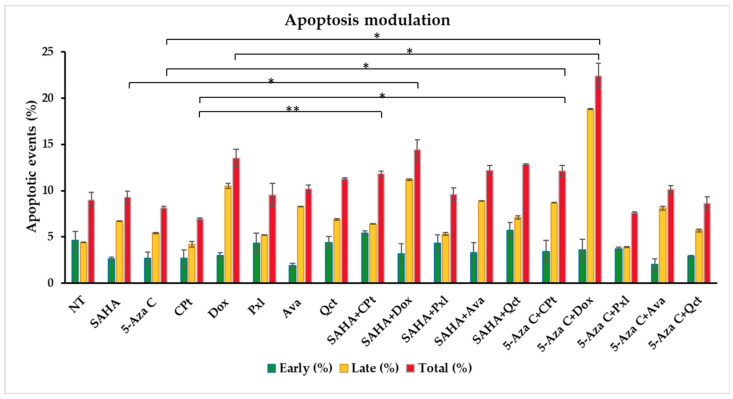
Impact of drug treatments on the induction of early and late apoptotic events in K1 human thyroid cancer cells, evaluated after Annexin-FITC/PI double staining and flow-cytometry data acquisition and analysis. Data were expressed as mean values ± SD (n = 3) (* *p* < 0.05, ** *p* < 0.01).

The single treatments with SAHA, 5-Aza-C, Pxl, Dox, Ava, and Qct induced increases in both late and total apoptosis, the highest effect being observed for SAHA, Dox, Ava, and Qct (Figure 4, Table S3, Figure S2).

When combined treatments were applied, the strongest effect was observed for SAHA and Dox, or 5-Aza-C and Dox that increased the late apoptosis levels to 11.2% and 18.8%, and total apoptosis to 14.4% and 22.4%, respectively. Also, the combinations of SAHA with CPt, Ava, and Qct, and 5-Aza-C with CPt or Ava induced increases in the total apoptosis levels (Figure 4, Table S3, Figure S2).

Statistical analyses showed significant differences between the levels of total apoptotic events when different single treatments were compared to their combinations. Significant increases were observed when cells were subjected to treatments with 5-Aza-C + Dox vs. Dox (*p* = 0.0139), 5-Aza-C + Dox vs. 5-Aza-C (*p* = 0.0255), 5-Aza-C + CPt vs. 5-Aza-C (*p* = 0.0333), SAHA + Dox vs. SAHA (*p* = 0.0286), SAHA + CPt vs. CPt (*p* = 0.0097), 5-Aza-C + CPt vs. CPt (p = 0.0292).

### 3.4. Modulation of Soluble Cytokines Released by Treated K1 Thyroid Cells

We have further evaluated the IL-6 and TNF-α cytokine release in the culture supernatants of 48 h drug-treated K1 thyroid cancer cells. To account for baseline secretion, values obtained after the quantification of secreted cytokines were calculated from the standard curves, expressed in pg/mL, and then results normalized versus non-treated cells (NT), considered as 100%, by reporting the concentrations in treated cell supernatants to control ones.

**Figure 5:**
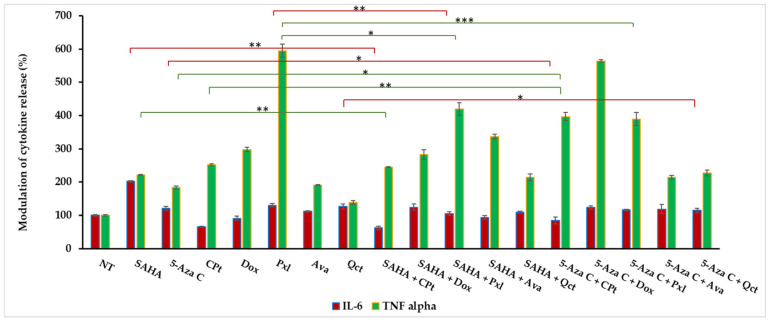
Evaluation of IL-6 and TNF-α levels in K1 thyroid cancer cell supernatants, released after single or combined SAHA or 5-Aza-C drug treatments. Levels of released cytokines by the non-treated cells (NT) were considered as 100% released. Data were expressed as mean values ± SD (n = 3); * *p* < 0.05, ** *p* < 0.01, *** *p* < 0.001. Horizontal red and green lines present the statistical data for IL-6 and TNF-alpha assays, respectively.

The modulation of IL-6 and TNF-α cytokine release was measured after 48 h treatments of K1 cells with previously chosen doses, i.e., either 50 µM of CPt, 0.5 μM for Dox, 0.5 µM of Pxl, 5 µM of SAHA or 5-Aza-C, 50 µM of Qct, and 20 µg/mL of Ava, and revealed higher levels of soluble cytokines across several treatment conditions, mostly in TNF-α secretion, compared to NT cells (Figure 5).

High increases in TNF-α released levels were measured both for single- and combined-drug treatments, the relative percentage values ranging from 138% for Qct to 595% for Pxl treatments of K1 cells. Treatments with SAHA and 5-Aza-C induced an n-fold increase in TNF-α release of 2.22 and 1.84, respectively, when compared to NT cells, while CPt, Dox, and Ava treatments led to n-fold increases of 2.52, 2.98, and 1.91, respectively. The combined treatments of SAHA with CPt, Pxl, Dox, and Ava induced higher levels of released TNF-α compared to SAHA single treatment, the relative percentage values reaching 244.6%, 419.8%, 282.7%, and 336%, respectively. When Qct was added to SAHA treatment, TNF-α release was comparable (214.2%). The same effects of increasing TNF-α release were also observed for 5-Aza-C combinations with CPt, Pxl, and Dox, reaching 396.9%, 389.3%, and 564.5% relative normalized values (Figure 5).

In contrast, levels of soluble IL-6 were lower than soluble TNF-α, both for single and combined treatments. Moreover, treatments with CPt, either single or combined with SAHA or 5-Aza-C, induced decreases in IL-6 release, until 65.4%, 62.3%, and 84.9%, respectively, while single SAHA treatments induced an increase to 201.8% compared to NT. When cells were treated with 5-Aza-C, we observed moderate increases for either single (121.4%) or combined treatments with Pxl, Dox, and Ava (ranging from 115.9% up to 123.7%) (Figure 5).

Statistical analyses showed significant differences between the soluble TNF-α levels, when different treatments and their combinations were compared: SAHA vs. SAHA + CPt (*p* = 0.0027), 5-Aza-C vs. 5-Aza-C + CPt (*p* = 0.0127), CPt vs. 5-Aza-C + CPt (*p* = 0.0203), Pxl vs. SAHA + Pxl (*p* = 0.0014), Pxl vs. 5-Aza-C + Pxl (*p* = 0.0003). We have also compared the results obtained for IL-6 release, and we observed significant decreases in IL-6 levels when the following treatments were applied: SAHA + CPt vs. SAHA (*p* = 0.0047), 5-Aza-C + CPt vs. 5-Aza-C (*p* = 0.0403), SAHA + Pxl vs. Pxl (p = 0.0058), and 5-Aza-C + Qct vs. Qct (*p* = 0.0494).

In addition, the experimental data were subjected to statistical analyses for the detection of potential correlations between the measured immune parameters. The correlation analyses revealed a negative significant correlation (*r* = −0.744, *p* < 0.05) between nuclei distribution in S phase and late apoptotic events, while a moderate direct correlation was observed (*r* = 0.405, *p* < 0.05) when compared to the early apoptosis percentages (Figure S3). Both G2M phase and proliferation index (S+G2M) directly corelated with early apoptotic events (*r* = 0.494, and *r* = 0.506), while with late apoptosis they demonstrated negative correlations (*r* = −0.293 and *r* = −0.577, respectively). TNF-α release negatively correlated with G0/G1 phase (*r* = −0.337) and positively correlated with the proliferation index (*r* = 0.337). In contrast, IL-6 levels positively correlated with G0/G1 phase (*r* = 0.243) and negatively correlated with S phase (*r* = −0.364) or proliferation index (*r* = 0.243) (Figure S3).

### 3.5. Quantification of Target lncRNAs

**Figure 6:**
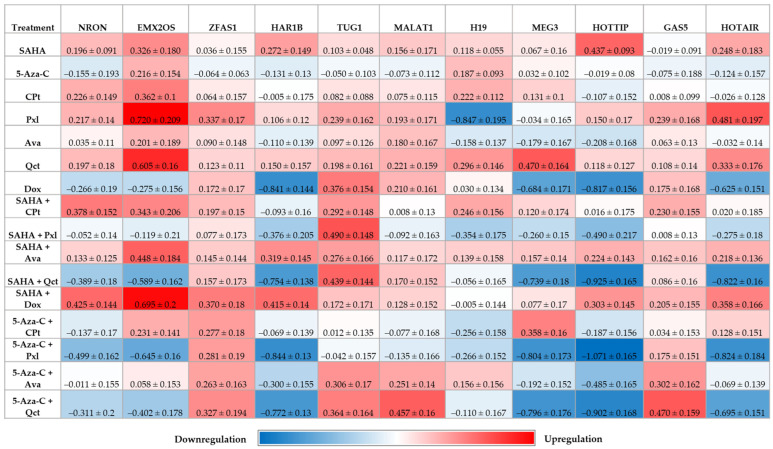
Heatmap showing log_10_-transformed values of double-normalized gene expression (relative to U6 and untreated controls) for selected lncRNAs across experimental models. The color gradient represents expression levels: red indicates upregulation, while blue indicates downregulation, with the intensity reflecting the magnitude of change.

The expression of selected lncRNAs and epigenetic modulators was analyzed across all treatment combinations, some lncRNAs displaying treatment-dependent changes in expression levels (Figure 6).

Treatment with four specific combinations: SAHA + Pxl, SAHA + Qct, 5-Aza-C + Pxl, and 5-Aza-C + Qct, induced the most pronounced effects on HAR1B, MEG3, HOTTIP, and HOTAIR leading to significant statistic downregulation of these lncRNAs (*p* < 0.0001 relative to NT, and to SAHA, *p* < 0.0001). HOTTIP showed the most pronounced downregulation, with n-fold values ranging from −0.902 to −1.071. Dox treatment alone produced similar effects on the aforementioned lncRNA species. 

ZFAS1, TUG1, MALAT1, and GAS5 exhibited a consistent expression pattern, showing only moderate or no increases across all treatments. In the case of ZFAS1, all combinations with 5-Aza-C yielded comparable n-fold expression values (ranging from 0.263 to 0.327), which were statistically significant compared to both NT (*p* < 0.0001) and 5-Aza-C alone (*p* < 0.0001). Notably, TUG1 (0.306 and 0.364 n-fold), MALAT1 (0.251 and 0.457 n-fold), and GAS5 (0.302 and 0.470 n-fold) showed increased expressions only in two treatment combinations: 5-Aza-C + Ava and 5-Aza-C + Qct, respectively.

Individual treatments had minimal impact overall, with a few exceptions. SAHA upregulated HOTTIP (0.437 n-fold, *p* < 0.0001 vs. NT while Qct increased both EMX2OS (0.605 n-fold) and MEG3 (0.470 n-fold) expression compared to NT (*p* < 0.0001). Pxl treatment elevated EMX2OS (0.720 n-fold) and HOTAIR (0.481 n-fold) levels, but reduced H19 expression (−0.847 n-fold, *p* < 0.0001 vs. NT).

**Figure 7:**
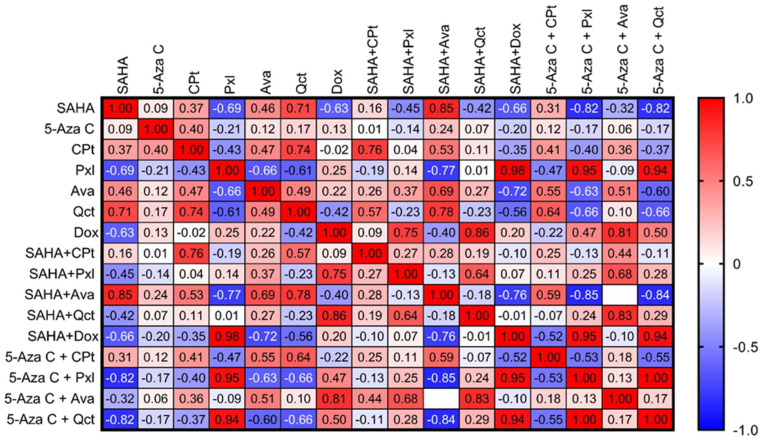
Correlogram illustrating *r* correlation coefficients for target gene expression profiles across various treatment conditions, with color gradients indicating the strength and direction of the relationship. The positive correlations are represented in red and negative in blue, with the intensity reflecting the magnitude of correlation.

Pearson correlation analysis revealed significant relationships in gene expression profiles across different treatment conditions (Figure 7). The analysis was performed using the expression values of all evaluated genes across the different treatments. The resulting Pearson *r* correlation coefficients represent the overall similarity in gene expression patterns between treatments.

Notably, SAHA treatment exhibited negative, inverse correlations with Pxl (*r* = −0.69, *p* = 0.005), Dox (*r* = −0.63, *p* = 0.012), SAHA + Dox (*r* = −0.66, *p* = 0.006), 5-Aza-C + Pxl (*r* = −0.82, *p* = 0.0003), and 5-Aza-C + Qct (*r* = −0.82, *p* = 0.0003). In contrast, SAHA showed strong positive correlations with Qct (*r* = 0.71, *p* = 0.003) and SAHA + Ava *(r* = 0.85, *p* = 0.093).

Gene expression in CPt-treated cells showed a strong correlation with Qct treatments (*r* = 0.74, *p* = 0.002), and an even stronger correlation with the SAHA + CPt combination (*r* = 0.76, *p* = 0.001).

Pxl treatment also demonstrated negative correlations with Ava (*r* = −0.66, *p* = 0.008), Qct (*r* = −0.61, *p* = 0.015), and SAHA + Ava (*r* = −0.77, *p* = 0.0008), but positive correlations with SAHA + Dox (*r* = 0.98, *p* < 0.0001), 5-Aza-C + Pxl (*r* = 0.95, *p* < 0.0001), and 5-Aza-C + Qct (*r* = 0.94, *p* < 0.0001).

Single treatments with Ava or Qct negatively correlated with SAHA + Dox (Ava: *r* = −0.72, *p* = 0.003; Qct: r = −0.56, *p* = 0.030), 5-Aza-C + Pxl (Ava: *r* = −0.63, *p* = 0.0116; Qct: *r* = −0.66, *p* = 0.0068), and 5-Aza-C + Qct (Ava: *r* = −0.60, *p* = 0.0190; Qct: *r* = −0.66, *p* = 0.0080). Conversely, both Ava and Qct were positively correlated with SAHA + Ava (*r* = 0.69, *p* = 0.0046, and *r* = 0.78, *p* = 0.0006, respectively). Additionally, Qct positively correlated with 5-Aza-C + CPt (*r* = 0.64, *p* = 0.011).

Dox treatment shared positive, direct correlations with combined regimens such as SAHA + Pxl (*r* = 0.75, *p* = 0.001), SAHA + Qct (*r* = 0.86, *p* < 0.0001), and 5-Aza-C + Ava (*r* = 0.81, *p* < 0.0001). No significant gene expression correlations were observed for cells treated solely with 5-Aza-C.

Among dual treatments, SAHA + Pxl correlated with SAHA + Qct (*r* = 0.64, *p* = 0.010) and 5-Aza-C + Ava (*r* = 0.68, *p* = 0.006), whereas SAHA + Ava negatively correlated with SAHA + Dox (*r* = −0.76, *p* = 0.001), 5-Aza-C + Pxl (*r* = −0.85, *p* = 0.0001), and 5-Aza-C + Qct (*r* = −0.84, *p* = 0.0001). SAHA + Dox strongly correlated with 5-Aza-C + Pxl (*r* = 0.95, *p* < 0.0001) and 5-Aza-C + Qct (*r* = 0.94, *p* < 0.0001) (Figure 7).

### 3.6. Analyses of Methylation-Related Genes, and Global DNA Methylation

**Figure 8:**
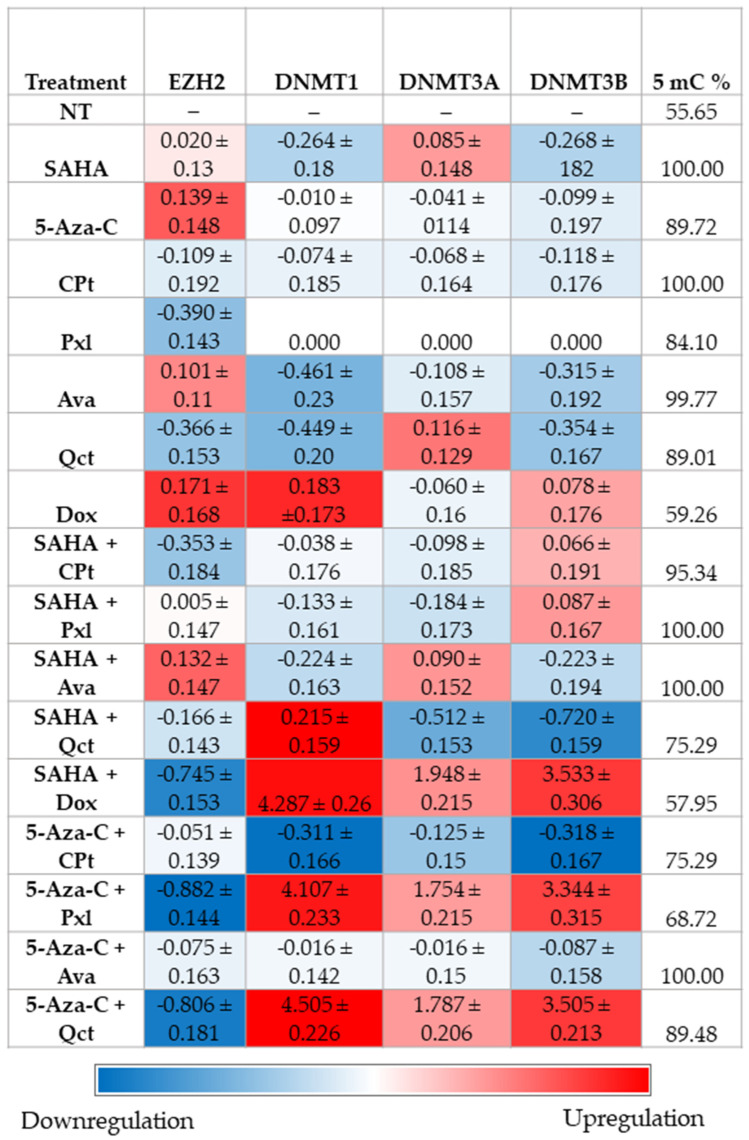
Heatmap showing mean log_10_-transformed values ± SD of double-normalized gene expression (relative to U6 housekeeping gene and untreated controls) for selected epigenetic modulators, and LINE global percentage methylation levels (5 mC%) across the experimental models. The color gradient represents expression levels: red indicates upregulation, blue indicates downregulation, with intensity reflecting the magnitude of change.

In terms of epigenetic modulation, three combined treatments significantly increased DNMT expression (Figure 8).

DNMT1 was strongly upregulated by SAHA + Dox (4.287-fold, *p* < 0.0001 vs. NT and SAHA), 5-Aza-C + Pxl (4.107-fold, *p* < 0.0001 vs. NT and 5-Aza-C), and 5-Aza-C + Qct (4.505-fold, *p* < 0.0001 vs. NT and 5-Aza-C). Likewise, DNMT3B expression was induced by SAHA + Dox (3.533-fold, *p* < 0.0001), 5-Aza-C + Pxl (3.344-fold, *p* = 0.0002), and 5-Aza-C + Qct (3.505-fold, *p* < 0.0001), compared to both NT and single-drug controls. Notably, no significant DNMT activation was observed with any single-agent treatment. Conversely, EZH2 expression was significantly downregulated by the same drug combinations that activated DNMTs. These expression modifications were accompanied by elevated LINE-1 methylation percentages, indicating a coordinated methylation activity and chromatin remodeling.

Global DNA methylation levels were expressed as mean percentage values and detailed in Figure 8. Linear regression analysis revealed a significant positive correlation between methylation levels and EZH2 expression (Y = 0.01178 × X − 1.258, *r* = 0.3255, *p* = 0.0210), and a significant negative correlation with DNMT3B expression (Y = −0.06049 × X + 6.288, *r* = 0.2628, *p* = 0.0423) (Figure 8, Figures S3 and S4).

## 4. Discussion

Thyroid cancer is the most common malignancy of the endocrine system, with approximately 820,000 diagnosed cases and 47,500 associated deaths reported worldwide by 2022 [46]. Although most thyroid cancers are manageable by surgical interventions, endocrine suppression therapy, and radioactive iodine radiotherapy, the mortality rate remains high in cases of advanced or iodine-refractory thyroid cancers. Therefore, elucidating the mechanisms underlying thyroid tumorigenesis and identifying reliable biomarkers for early diagnosis and use in targeted therapy remain key priorities in current oncological research. Accumulating scientific evidence indicates that lncRNAs are aberrantly expressed in various human cancers and play critical roles in regulating key cellular processes, including tumor growth, apoptosis, invasion, metastasis, and response to chemotherapy [47,48,49].

Our previous studies regarded changes in DNA methylation in thyroid cancer patients. We evaluated the degree of promoter methylation for several markers associated with thyroid neoplasms and showed their relationship with thyroid oncogenesis. We found new characteristics of thyroid tumors, such as methylation of TP73, WIF1, and PDLIM4 TSGs, which can contribute to thyroid neoplasia. Furthermore, we observed a significant correlation between RET/PTC rearrangements or BRAF V600E mutation with TIMP3 and CDH13, RARB methylation. Therefore, we concluded that TSGs promoter hypermethylation is a hallmark of cancer and a test that uses methylation quantification method is suitable for diagnosis and prognosis of thyroid cancer [44,50]. Further studies were focused on the connection between global and local DNA methylation status, and the assessment of levels of DNA methylation regulators (TET family and DNMT1) in thyroid tumors. Results suggested that TET1/TET2 gene expression and DNA methylation may serve as potential diagnostic tools for thyroid neoplasia. In addition, in vitro preliminary studies performed on K1 cell line treated with 5-Aza-C demethylating agent showed demethylation effects, especially upon TET2 gene, opening perspectives for thyroid cancer therapy [44,50]. Our preliminary results showed that 5-AzaC can induce cytotoxicity, modulated cell cycle phases’ distribution, time- and dose-dependent, increased apoptosis of human glioma U-87 MG [45] and thyroid K1 tumor cell lines [44], all these data being correlated with the differences observed in the methylation pattern [44,45]. All these results prompted us to expand our studies on other factors that could contribute to epigenetic remodeling in thyroid cancer, such as lncRNAs.

Our present study reveals new insights into the epigenetic mechanisms underlying thyroid carcinoma, particularly the role of lncRNAs, examining their expression in response to single and combined treatments with the HDAC inhibitor SAHA, and the DNA methyltransferase inhibitor 5-Azacytidine, conventional chemotherapeutic agents (Carboplatin, Doxorubicin, or Paclitaxel), anti-angiogenic monoclonal antibody Avastin, or the natural compound Quercetin, and their combinations. All tested compounds induced cell lysis in K1 human thyroid cancer cells in a dose and time-dependent manner, the strongest cytotoxic effects being observed after 48 h of treatment.

Further, the impact of SAHA or 5-Aza-C epigenetic drugs, used either alone or in combination with other therapeutic agents, was evaluated on the distribution of K1 cell populations across different phases of the cell cycle Nuclei increases in G0/G1 phase were observed following either single treatments with SAHA or 5-Aza-C or their combinations with Dox or Ava. Moreover, decreases in S phase were observed with SAHA or 5-Aza-C administered as single treatments, and the decreases were even stronger when the epigenetic drugs were combined with Dox. On the other hand, single treatments with CPt or Pxl increased S phase, but their combination with 5-Aza-C decreased the rate of S phase, even if it remained higher compared with the NT control. A strong increase in G2M phase was observed for the treatments with these drug combinations.

Moreover, total and late apoptosis were induced in cells treated with single treatments of SAHA, 5-Aza-C, Pxl, Ava, and Qct, while the combined treatments enhanced the apoptotic effects, with SAHA + Dox, and 5-Aza-C + Dox treatments inducing the strongest increase in late and total apoptosis.

Secreted by activated immune cells, cytokines serve as central mediators of immune signaling. They play essential roles in orchestrating immune and inflammatory responses, with key cytokines such as TNF-α and IL-6 being critical in processes like macrophage activation, antigen presentation, and regulation of inflammation. Due to their broad impact on physiological and pathological processes, monitoring cytokine activity is vital, as their effects can be either beneficial or detrimental, depending on their concentration and the microenvironmental context [51,52]. Single and combined SAHA or 5-Aza-C treatments induced specific variations in cytokine secretion. The statistical analyses showed significant differences between the soluble TNF-α levels following treatments with SAHA or 5-Aza-C combinations with Cpt when compared with single treatments. The same combined treatments induced significant decreases in IL-6 levels, but also for SAHA + Pxl vs. Pxl and 5-Aza-C + Qct vs. Qct.

Regarding lncRNA expression, the current study revealed a significant downregulation of HAR1B, MEG3, HOTTIP, and HOTAIR expression following combined treatments with SAHA + Pxl, SAHA + Qct, 5-Aza-C + Pxl, and 5-Aza-C + Qct, indicating the involvement of these epigenetic modulators in the suppression of oncogenic lncRNAs in thyroid cancer cells. To date, HAR1B has not been specifically associated with thyroid cancer in existing literature; therefore, the present study is the first to report its expression following various treatments in the K1 human thyroid cancer cell line. According to Waters et al., HAR1A and HAR1B can act either as oncogenes or tumor suppressors in a cancer type-dependent manner, with their expression levels having potential as prognostic biomarkers, particularly in gliomas and oral carcinomas [53]. Inhibition of HAR1B expression has been associated with enhanced migration and invasion in gliomas, and with increased resistance to pazopanib in sarcoma [53,54].

Dadafarin et al. reported that MEG3 is downregulated in papillary thyroid carcinoma and associated with lymph node metastasis. MEG3 suppresses cancer cell invasion and migration by inhibiting *Rac1* expression via its 3′UTR. An inverse correlation between MEG3 and *Rac1* was observed, supporting the role of MEG3 as a tumor suppressor in thyroid carcinoma [55,56,57]. In our study, MEG3 expression was upregulated only after treatment with Qct alone and in combination of 5-Aza-C and CPt, both interventions with potential to counteract oncogenic processes.

Another investigated lncRNA, HOTTIP, promotes tumorigenesis by modulating cell proliferation, invasion, apoptosis, and metastasis. Its overexpression has been documented in multiple cancers, including hepatocellular, breast, gastric, pancreatic, and esophageal squamous cell carcinomas, where it acts as an oncogene [33,58,59,60,61]. According to our findings, the most pronounced decrease in HOTTIP expression was observed following treatment with two 5-Aza-C combinations, highlighting the significant role of DNA methylation in its regulation.

Regarding another investigated lncRNA, HOTAIR is differentially expressed in both tissues and plasma of thyroid cancer (TC) patients compared to controls, with higher levels associated with tumor aggressiveness and progression. In vitro HOTAIR functions as an oncogene, as its knockdown inhibited TC cell proliferation and invasion [62]. Despite extensive evidence supporting HOTAIR as a diagnostic and prognostic marker in many solid tumors, only one clinical trial has investigated its role in thyroid cancer (NCT03469544), highlighting the need for in-depth studies to validate its potential and utility as a biomarker in predicting response to therapy and guiding patient management [63]. Our results revealed the potential of four compound combinations, suggesting their efficacy in downregulating oncogenic lncRNAs such as HOTAIR, thus offering promising directions for targeted epigenetic therapy in thyroid cancer.

Zinc finger antisense 1 (ZFAS1) has gained recognition as a key oncogenic driver, playing a significant role in the progression of various cancers. Notably, studies have consistently reported increased expression of ZFAS1 in thyroid cancer (TC) cells, further highlighting its tumor-promoting functions [27]. Han et al. revealed in their cellular experiments that ZFAS1 knockdown suppressed proliferation by inducing cell cycle arrest—a key anticancer mechanism. Since disrupted cell cycle regulation promotes tumorigenesis by altering proliferation–death balance, and given previous links between lncRNAs (e.g., GAS5, Linc00152) and cell cycle control, their findings that ZFAS1 depletion increased G1 phase and decreased S phase levels reinforce its oncogenic role in human cancers [64]. Our experiments showed that most treatments did not significantly affect ZFAS1 expression levels; however, a slight upregulation was observed in all combinations involving 5-Aza-C.

A similar pattern was observed for TUG1 expression, with slight increases occurring in almost all combinations involving SAHA. According to the findings of Lei et al., TUG1 functions as a potential oncogene, with its expression being significantly upregulated in thyroid cancer tissues. TUG1 has been shown to enhance cell proliferation, migration, and epithelial–mesenchymal transition (EMT) in thyroid cancer cells. Mechanistically, TUG1 appears to exert its effects through the miR-145/ZEB1 signaling pathway, highlighting its potential relevance in the prognosis and therapeutic targeting of thyroid cancer [28].

MALAT1 (Metastasis-Associated Lung Adenocarcinoma Transcript 1) is a long noncoding RNA involved in the regulation of the cell cycle and cell migration. Although MALAT1 dysregulation has been documented in numerous malignancies—such as lung, uterine, cervical, breast, colon, pancreatic, gastric, renal, bladder, and bone cancers—our study found that most treatments had little or no effect on MALAT1 expression, with only minor increases observed in some cases [65].

In a study by Gu, reduced expression of EMX2OS was identified and suggested as a potential prognostic biomarker for poor recurrence-free survival (RFS) in classical papillary thyroid carcinoma (PTC) [26]. Our results showed that EMX2OS expression was both upregulated and downregulated following treatments, with the most notable effects observed in treatments that increased its expression (Pxl, Qct, and SAHA + Dox).

Pearson correlation analysis revealed key gene expression relationships under treatment conditions, offering insights into drug mechanisms and interactions. A strong correlation of CPt with the SAHA + CPt combination was observed (*r* = 0.76.), highlighting the enhanced effect of CPt when used in combination therapy. A similar effect was observed for Pxl which significantly correlated with 5-Aza-C + Pxl combination treatment. Additional strong positive correlations were observed between Pxl and both SAHA + Dox, and 5-Aza-C + Qct treatments, suggesting common or overlapping molecular pathways.

Interestingly, correlation analysis of gene expressions after SAHA treatment exhibited negative values, either with itself and several of its combinations, suggesting potential antagonistic interactions—also evident in its negative correlations with 5-Aza-C + Pxl and 5-Aza-C + Qct treatments. Two additional strong positive correlations were observed between SAHA + Dox and both 5-Aza-C + Pxl and 5-Aza-C + Qct treatments, suggesting that these combinations may share common mechanisms of epigenetic or apoptotic regulation, enhanced by dual modulation of histone deacetylation and DNA methylation. The absence of significant correlations for 5-Aza-C alone or in combination suggests that its effects may be context-dependent, possibly relying on specific cellular states or requiring synergistic partners to exert measurable changes in gene expression.

Global hypomethylation, a hallmark of tumor cells, contributes to chromosomal instability and altered gene expression, often acting through cis-regulatory effects near hypomethylated regions. Given that LINE-1 elements can exhibit locus-specific methylation patterns, their roles in cancer may vary depending on the genomic context. In our study, treatments such as SAHA + Dox, 5-Aza-C + Pxl, and 5-Aza-C + Qct resulted in increased global methylation. This was accompanied by a positive correlation between EZH2 downregulation and LINE-1 methylation levels, as well as an inverse correlation between DNMT3B expression and global LINE-1 methylation levels, suggesting that these treatments may restore epigenetic balance by repressing oncogenic pathways (via EZH2) and reducing DNMT3B-associated demethylation, thereby contributing to genome stabilization in K1 human thyroid cancer cells. Furthermore, the applied treatments appear to counteract the global pattern of hypomethylation that typically characterizes cancer cells. Future studies should focus on exploring specific methylation patterns, particularly at the level of key gene promoters, which are often marked by hypermethylation [66].

## 5. Conclusions

The combined treatments SAHA + Dox, 5-Aza-C + Pxl, and 5-Aza-C + Qct significantly increased DNMT1 and DNMT3B expression, and simultaneously reduced EZH2 expression —an effect not observed with any single-agent treatment, suggesting a coordinated epigenetic modulation that may contribute to their enhanced therapeutic potential. In particular, the combined use of HDAC inhibitors (SAHA) or 5-Aza-C with therapeutic agents demonstrated a strong potential for reactivating silenced tumor suppressor genes, with methylation changes observed in vitro. These promising results lay the foundation for further validation in animal models and may facilitate clinical translation, especially given the existing approval of some HDAC inhibitors for human therapy.

These findings add to the growing evidence that combinatorial strategies involving epigenetic modulators and chemotherapeutic agents can help overcome or reverse the drug resistance and improve the clinical outcomes and quality of life in patients with thyroid cancer. The distinct cytokine responses observed with single versus combined treatments suggest the potential for personalized approaches based on cytokine modulation, emphasizing the need to further explore the mechanisms underlying these effects to optimize therapeutic strategies in thyroid cancer.

## Figures and Tables

**Table 1:** IC50 and IC75values that induced K1 cell lysis.

Compound/ Time	IC50 (µM)		IC75 (µM)	
	24 h	48 h	24 h	48 h
CPt	187.23 ± 7.43	126.44 ± 8.74	≥200	≥200
Dox	6.82 ± 0.22	1.89 ± 0.09	26.51 ± 1.93	22.06 ± 2.15
Pxl	0.51 ± 0.03	0.05 ± 0.01	2.62 ± 0.21	2.13 ± 0.12
SAHA	17.49 ± 1.01	15.02 ± 1.96	38.93 ± 3.41	36.21 ± 2.56
Qct	92.02 ± 6.32	79.43 ± 5.24	175.83 ± 9.87	153.59 ± 8.75

IC50 and IC75 values represent the concentrations of the tested compounds needed to inhibit 50% and 75%, respectively, of the cell growth, presented as mean values ± SD (n = 3).

## Data Availability

The data generated or analyzed during this study are included in the article and Supplementary Materials. Further inquiries can be directed to the corresponding author(s).

## Supplementary Materials

The following supporting information can be downloaded at https://www.mdpi.com/article/10.3390/cimb47100863/s1.
