# Supplementary material for: Epigenetic Remodeling in Thyroid Cancer: New Dimensions of Targeted Therapy Through lncRNA Modulation
Source: Curr Issues Mol Biol. 2025 Oct 18;47(10):863. doi: 10.3390/cimb47100863 (PMC12563275; doi:10.3390/cimb47100863)
Supplement: Supplementary file 1 [file cimb-47-00863-s001.zip › S1, S2, S3 supplementary tables_.pdf]

Table S1. Primer sequences for the investigated target genes

Target	Primer sequence	Reference
NRON	F: CATGGCGACGGCAAAATCAT	[45]
	R: AACCCCCAAACCTTCCGATG	
EMX2OS	F: CTTGCACCAACCCTTTTCCG	[45]
	R: CAGCCTAGACCACCTACCCT	
ZFAS1	F: ATTGTCCTGCCCGTTAGAGC	[45]
	R: ACTTCCAACACCCGCATTCA	
HAR1B	F: ACGTCTCCTCCGTTTCATGC	[45]
	R: TCAGACCTGGTTGCAGAGTG	
TUG1	F: CGACTGAGCAAGCACTACCA	[45]
	R: CTCAGCAATCAGGAGGCACA	
HOTAIR	F: GGTAGAAAAAGCAACCACGAAGC	[45]
	R: ACATAAACCTCTGTCTGTGAGTGCC	
GAS5	F: CTTCTGGGCTCAAGTGATCCT	[45]
	R: TTGTGCCATGAGACTCCATCAG	
MEG3	F: ATCCTGCTGGCAACTCCAAG	[45]
	R: GCGTGCCTTTGGTGATTCA	
HOTTIP	F: ACTTTCTGGCCGTTTCACCA	[45]
	R: CAGGGGCCTAGAACCCTACT	
MALAT1	F: AAAGCAAGGTCTCCCCACAAG	[45]
	R: GGTCTGTGCTAGATCAAAAGGCA	
U6	F: CTCGCTTCGGCAGCACATATACT	[45]
	R: ACGCTTCACGAATTTGCGTGTC	
H19	F: TGCTGCACTTTACAACCACTG	[45]
	R: ATGGTGTCTTTGATGTTGGGC	
EZH2	F: TGCAACACCCAACACTTATAAGCGG	[45]
	R: CCTTTGCTCCCTCCAAATGCTGGT	
DNMT1	F: GCGGTATACCCACCATGACA	[45]
	R: AGGCTTTGCCGGCTTCC	
DNMT3A	F: CTGACAGAGGCACCGTTCAC	[45]
	R: TATCGTGGTCTTTGGAGGCG	
DNMT3B	F: CCAACAACACGCAACCAGTG	[45]
	R: CGTCTTCGAGTCTTGTTCTCGTA	

Table S2. Cell cycle phases distribution in treated and non-treated K1 thyroid cancer cells.

**Drug treatment**	**Cell cycle phases**			**Proliferation index**
	**G0/G1 (%)**	**S (%)**	**G2M (%)**	**S+G2M (%)**
**NT**	71.41	19.7	8.89	28.59
**SAHA (5 μM)**	89.69	3.68	6.63	10.31
**5-Aza-C (5 μM)**	92.57	3.71	3.72	7.43
**CPt (50 μM)**	36.66	54.25	9.09	63.34
**Dox (0.5 μM)**	91.85	2.28	5.87	8.15
**Pxl (0.5 μM)**	44.56	47.44	8.00	55.44
**Ava (20 μg/ml)**	90.16	4.65	5.18	9.83
**Qct (50 μM)**	71.59	14.18	14.24	28.42
**SAHA + CPt**	46.99	45.01	8.00	53.01
**SAHA + Dox**	89.97	2.43	7.60	10.03
**SAHA + Pxl**	41.68	50.32	8.00	58.32
**SAHA + Ava**	93.28	3.45	3.27	6.72
**SAHA + Qct**	67.28	14.26	18.47	32.73
**5-Aza-C + CPt**	24.71	30.05	45.25	75.30
**5-Aza-C + Dox**	88.42	2.27	9.30	11.57
**5-Aza-C + Pxl**	10.49	24.92	64.59	89.51
**5-Aza-C + Ava**	89.42	3.76	6.82	10.58
**5-Aza-C + Qct**	74.15	7.26	18.59	25.85

Table S3. Apoptosis modulation in treated and non-treated K1 thyroid cancer cells.

Treatments	Apoptotic events (%)		
	Early apoptosis	Late apoptosis	Total apoptosis
NT	4.6	4.4	9
SAHA (5 μM)	2.6	6.7	9.3
5-Aza-C (5 μM)	2.7	5.4	8.1
CPt (50 μM)	2.7	4.2	6.9
Dox (0.5 μM)	3	10.5	13.5
Pxl (0.5 μM)	4.3	5.2	9.5
Ava (20 μg/ml)	1.9	8.3	10.2
Qct (50 μM)	4.4	6.9	11.3
SAHA + CPt	5.4	6.4	11.8
SAHA + Dox	3.2	11.2	14.4
SAHA + Pxl	4.3	5.3	9.6
SAHA + Ava	3.3	8.9	12.2
SAHA + Qct	5.7	7.1	12.8
5-Aza-C + CPt	3.4	8.7	12.1
5-Aza-C + Dox	3.6	18.8	22.4
5-Aza-C + Pxl	3.7	3.9	7.6
5-Aza-C + Ava	2	8.1	10.1
5-Aza-C + Qct	2.9	5.7	8.6
