# Supplementary figures and images for: Epigenetic Remodeling in Thyroid Cancer: New Dimensions of Targeted Therapy Through lncRNA Modulation
Source: Curr Issues Mol Biol. 2025 Oct 18;47(10):863. doi: 10.3390/cimb47100863 (PMC12563275; doi:10.3390/cimb47100863)

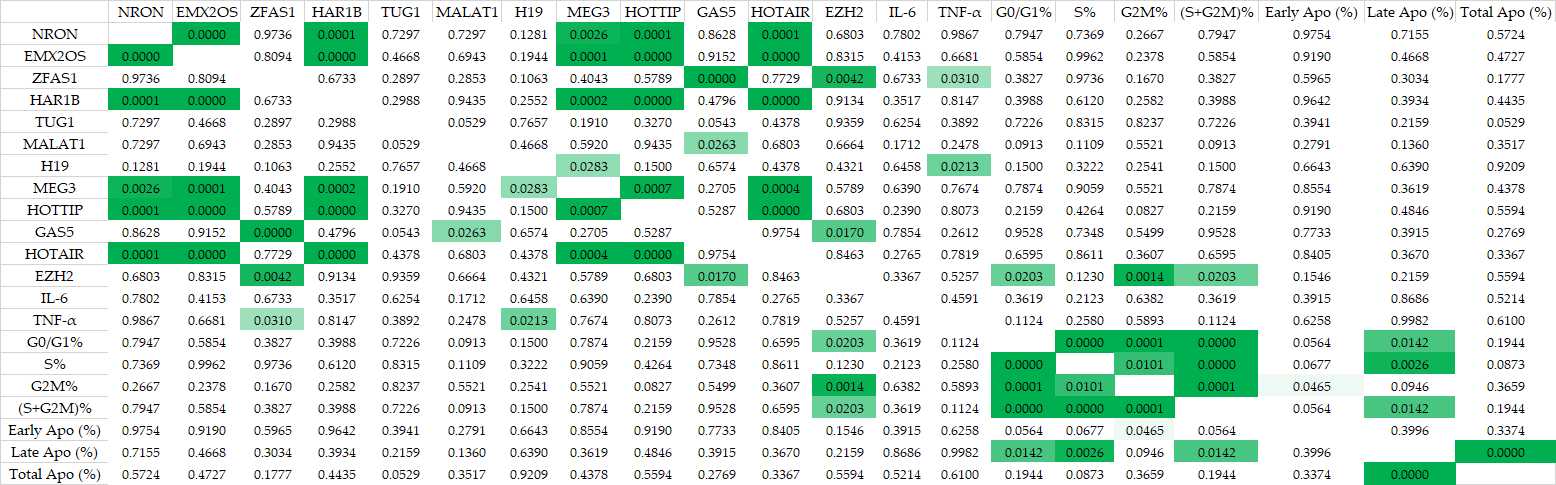

Supplement: Supplementary file 1 [file cimb-47-00863-s001.zip › Fig S4.tif]

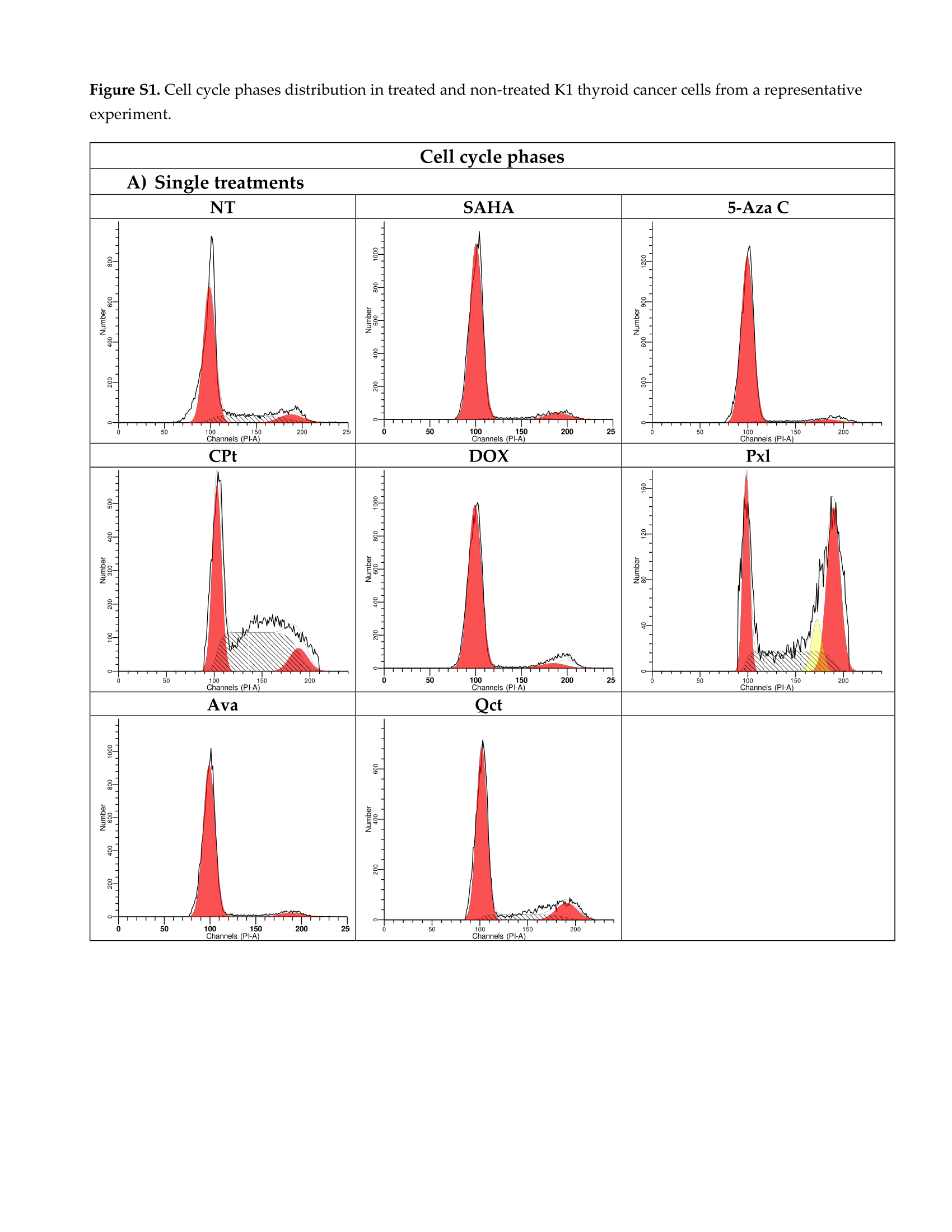

Supplement: Supplementary file 1 [file cimb-47-00863-s001.zip › Figure S1_K1 cell cycle histograms.tiff]

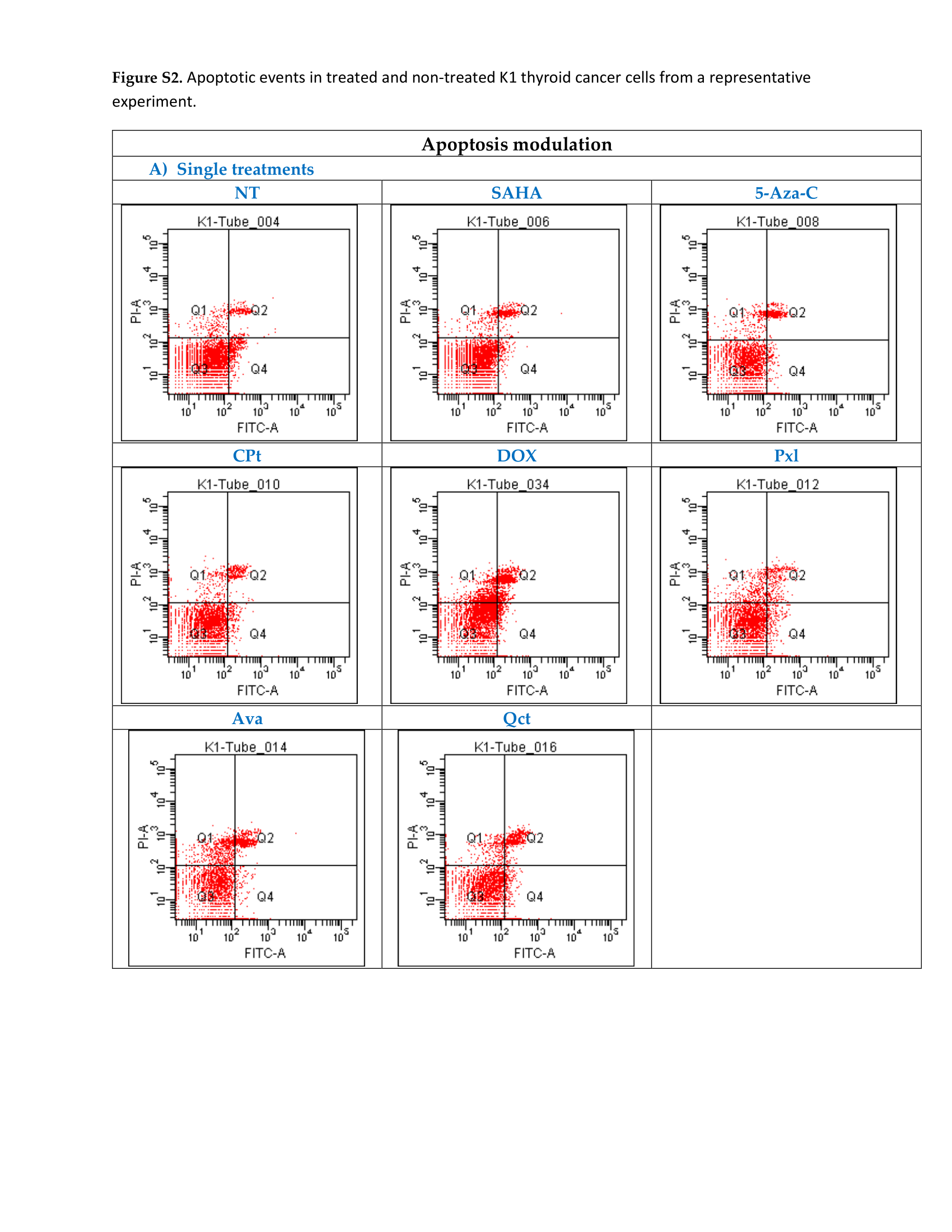

Supplement: Supplementary file 1 [file cimb-47-00863-s001.zip › Figure S2_K1 apoptosis dot plots.tiff]

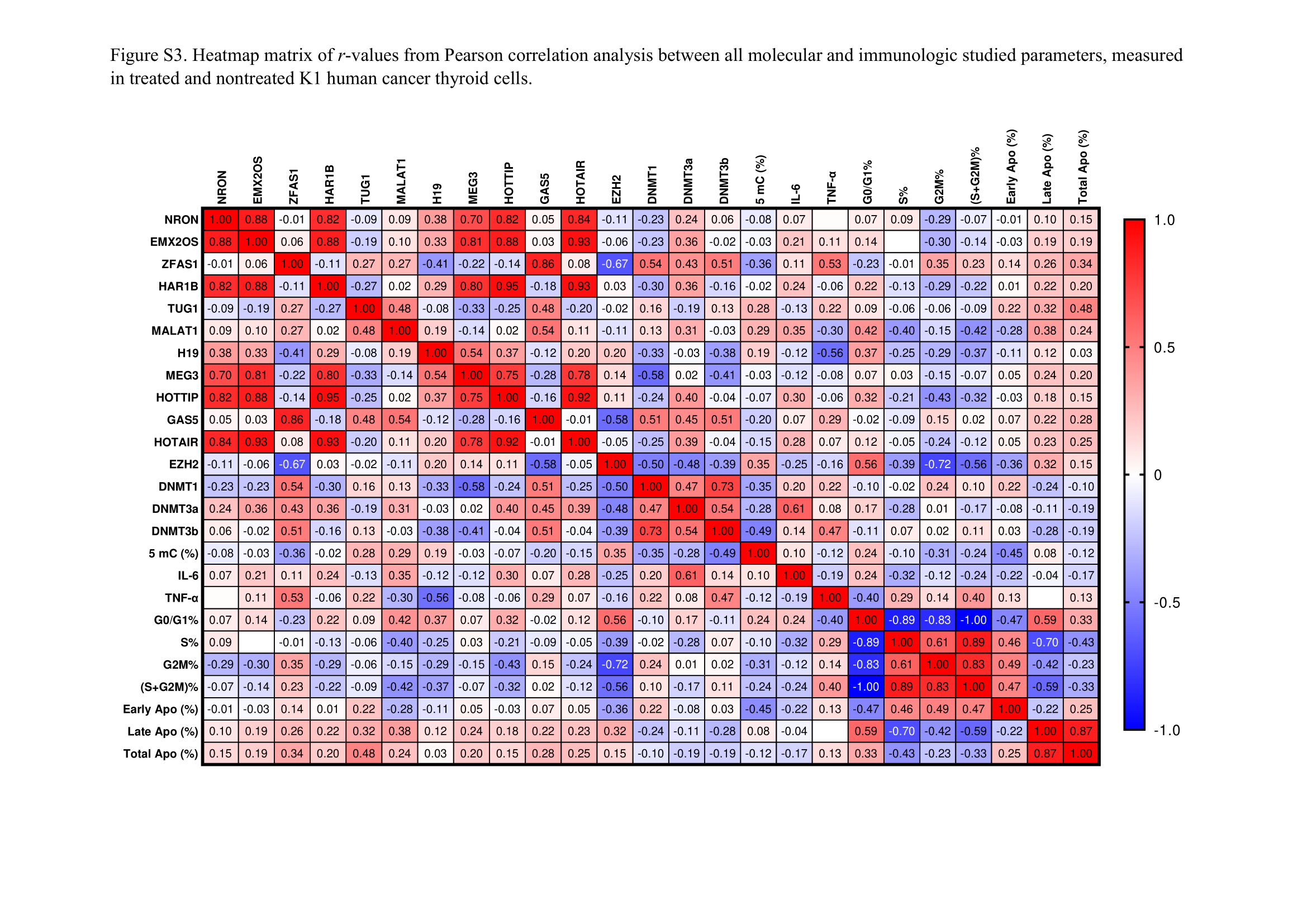

Supplement: Supplementary file 1 [file cimb-47-00863-s001.zip › Figure S3 Heatmap correlation r values all parameters.tiff]
